# The Woofer-Type Piezo-Actuated Microspeaker Based on Aerosol Deposition and Metal MEMS Process

**DOI:** 10.3390/mi16030353

**Published:** 2025-03-20

**Authors:** Wei-Ting Shih, Wan-Hsin Tsou, Dejan Vasic, François Costa, Wen-Jong Wu

**Affiliations:** 1 Department of Engineering Science and Ocean Engineering, National Taiwan University, Taipei 10617, Taiwan; 2 CY Cergy Paris University, 95000 Cergy, France; 3 SATIE, ENS Paris-Saclay, 91190 Gif-sur-Yvette, France; 4 University Paris-Est Créteil, INSPE, 94000 Créteil, France; 5 University Paris-Saclay, ENS Paris-Saclay, CNRS, SATIE, 91190 Gif-sur-Yvette, France

**Keywords:** piezoeletric, MEMS speaker, piezo stacking, bimorph, packaging

## Abstract

In this study, we present two configurations of piezo-actuated microspeakers, which were fabricated by combining a self-developed aerosol deposition method with the metal MEMS microfabrication process. The stainless steel used was structurally designed to enhance the displacement amplitude of the speaker, which is related to its sound pressure level. The two packaged speakers were measured using the IEC 60318-4 standard. The package around the speaker contains a printed circuit board with the dimensions in 20.0 mm × 13.0 mm × 3.0 mm. In an enclosed field test, the bimorph single-layer (BSL) configuration reached sound levels of 98.4 dB and 92.4 dB using driving voltages of 30 Vpp and 15 Vpp at 1 kHz, respectively; however, the bimorph multi-layer (BML) configuration reached higher levels of 108.2 dB and 102.2 dB under the same conditions.

## 1. Introduction

The continuous development of wearable electronics, true wireless stereo (TWS) headsets, smart phones, and smart controllers brings with it an enormous demand for miniaturized speakers. According to market research conducted by Yole Group, the revenue of the MEMS acoustic industry reached USD 15.3 trillion in 2020 [1]. Speakers accounted for the largest share, 59%, of the total market revenue from this industry, while audio CODEC products represented 13% and microphones accounted for 9%, making them the third most profitable. Yole Group also predicts that the total market revenue of the MEMS acoustic industry will reach USD 21.4 trillion, with a compound annual growth rate (CAGR) of 5.8% [2]. These numbers clearly indicate the potential demand for these electronics in the future.

With progression in the miniaturization of electronic devices, the trade-off between speaker performance and dimension becomes the issue. In particular, the displacement amplitude of the speaker’s diaphragm is related to the output performance of its low-frequency waves. Microspeakers can be categorized into four categories based on the differences in the driving mechanisms they use. The most common is the moving-coil-type speaker [3,4,5,6,7,8]. By alternating the direction of the current in the coil, the diaphragm is actuated by the changes in the magnetic force between the coil and the permanent magnet that surrounds it. The output power and the displacement for this type of speaker are related to the driving force, which is correlated to the diameter of the coil and the size of the permanent magnet. Clearly, these two restrictions indicate a trade-off between product miniaturization and output performance. Electrostatic-type speakers are realized by inserting a conductive diaphragm between biased electrodes [9,10,11,12,13,14]. When a bias voltage is applied to the diaphragm, the diaphragm is polarized and then moves due to electrostatic forces. This type of speaker usually requires a high driving voltage to attract or repel the diaphragm quickly enough to obtain adequate sound pressure level performance. Thermal acoustic speakers convert heat from a conductive material into acoustic energy [15,16,17,18]. One group of candidate materials for these speakers is carbon-based materials such as graphene and carbon nanotubes. Graphene has a low thermal capacitance per area unit and excellent thermal conductivity. However, the selection of the substrate used for this type of speaker is quite critical, as the thermal conduction loss between the substrate and the diaphragm causes a low output performance.

The last type is the piezoelectric microspeaker, which has only recently been developed by research groups and commercial companies. For the sake of clarity, in this paper we divide the devices we have made into two subtypes. One type of device functions using the MEMS process alone. Its basic design has a sandwich structure, and there is no plastic diaphragm in the device; instead, the whole device vibrates to generate the acoustic wave. That is, the diaphragm part of the speaker is the whole device. The main advantage compared to traditional moving-coil-type microspeakers is that this eliminates the bulky coil and magnet that are used to actuate the diaphragm. As a result, this type of device can become more compact than other speakers [19,20,21,22,23,24,25,26]. In contrast, the other type of device only uses a piezoelectric film as its actuator. The acoustic wave is generated by the diaphragm, which is bonded with the actuator [27]. This type of device is similar to those with a traditional moving-coil structure, and its design is highly flexible. Rather than considering the whole structure to be the actuator and diaphragm at the same time, this design can focus on the performance of the actuator.

For years, a large drawback in the coverage of microspeakers has been their low SPL performance in their low-frequency range. The displacement of the diaphragm is critical to enhancing the SPL in the low-frequency range. To obtain a higher displacement amplitude for the diaphragm while maintaining a low driving voltage, Cho et al. [21] used polyimide as their diaphragm material, which has a low Young’s modulus value, and drove the diaphragm using a sol–gel-deposited PZT film. Packaged with a printed circuit board, their speaker has a sound pressure level (SPL) performance of 78 dB at 1 kHz. To achieve a higher SPL performance and lower distortion, Kim et al. [22] inserted a silicon buffer layer between the piezoelectric layer and the substrate. This buffer layer reduces the first resonant frequency to 492 Hz rather than 1.2 kHz and achieves an SPL of up to 94 dB and a total harmonic distortion (THD) that is less than 15% of that in their previous work. Cheng et al. [20] designed a spring-like structure with a dual-electrode driving mechanism to improve the SPL performance of a microspeaker. This structure divides the volume of the diaphragm into several parts, meaning that the actuated force can be confined to a small volume. Under fixed dimensions, with a fixed driving voltage, and at 1 kHz, the maximum displacement of the film was improved from 2.32 µm to 4.75 µm. From this, we can categorize the following areas of improvement for piezoelectric microspeakers: substrate selection and pattern design, diaphragm selection, the deposition quality of the piezoelectric film, and electrode distribution.

The remainder of this paper is organized as follows: Section 2 provides the acoustic theory of the relationship between the sound pressure level performance of a device and its configuration, using the results of simulations performed in COMSOL software (version 6.0). Section 3 describes the process of fabricating our piezoelectric microspeaker. Section 4 presents the results achieved with the bimorph single-layer configuration and bimorph multi-layer configuration during simulations and in real devices measured under IEC 60318-4 standard [28] enclosed field conditions, and finally the conclusions of this study are presented in Section 5.

## 2. Theoretical Analysis and Simulation

### 2.1. Free-Field Acoustic Analysis

**Figure 1:**
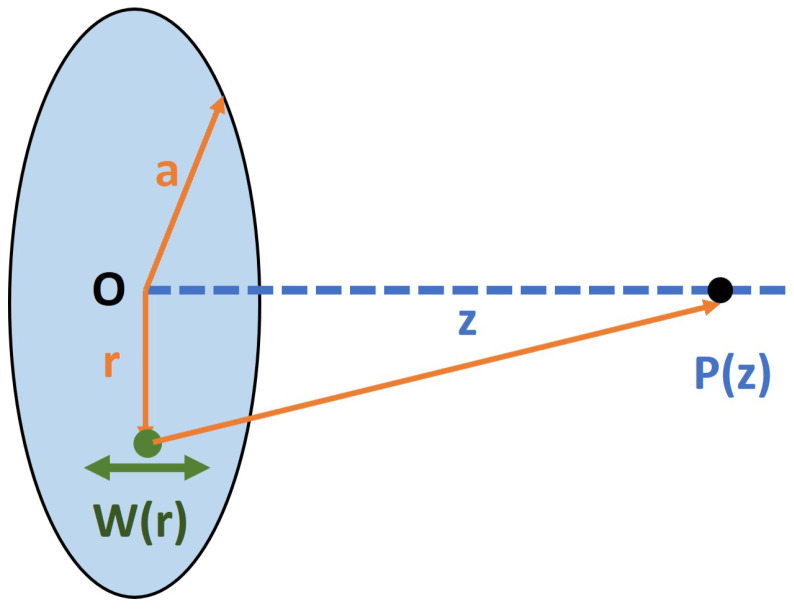
A schematic view of the piston-like motion of speakers used for sound pressure level calculations.

To quantify the performance of the devices, we considered their sound pressure level (SPL) and total harmonic distortion (THD), which are the two main indices used in the acoustic industry. The microspeaker can be simulated as a piston-like motion, as shown in Figure 1. The pressure level at point *z*, P(z), can be derived from Equation (1):

(1) $$P\left(z\right)=\rho {\left(2\pi f\right)}^{2}\int_{0}^{a}\frac{w(r)}{\sqrt{z^{2}+r^{2}}}rdr$$

where ρ stands for the density of air, *f* for the vibration frequency, and *a* for the radius of the diaphragm. By simplifying the movement of a diaphragm with an infinite radius to the motion of a piston, the effective pressure level $P_{e}\left(z\right)$ can be derived from Equation (2):

(2) $$P_{e}\left(z\right)=\frac{P(z)}{\sqrt{2}}=\frac{\sqrt{2}\pi \rho Swf^{2}}{z}$$

where *S* stands for the area of the diaphragm, *f* for the vibration frequency, and *w* for the amplitude of the diaphragm’s displacement. Following the calculation of the effective pressure level, the sound pressure level (SPL) can be derived from Equation (3):

(3) $$SPL\left(dB\right)=20log_{10}\left(\frac{P_{e}}{P_{ref}}\right)=10log_{10}{\left(\frac{P_{e}}{P_{ref}}\right)}^{2}$$

where $P_{ref}$ stands for the referenced acoustic pressure level, which is 20 µPa in open-field conditions.

### 2.2. Enclosed-Field Acoustics Analysis

To simulate the use of the speakers in headphones, the pressure level conditions should change from a free field to an enclosed field. Equation (4) describes the new pressure level calculation.

(4) $$P=\frac{P_{0}\times \left(\frac{C_{p}}{C_{v}}\right)}{V}\cdot S\cdot x$$

where *P* stands for the output pressure, $P_{0}$ for 1 atmospheric pressure, $C_{p}$ for the specific heat capacity of the air at constant pressure, $C_{v}$ for the specific heat capacity of the air at constant volume, *V* for the enclosed field acoustic volume, *S* for the area of the diaphragm, and *x* for the amplitude of the diaphragm’s displacement.

### 2.3. Simulation of Piezoelectric Microspeaker

This study uses COMSOL multiphysics simulation software to verify the design parameters of the microspeakers we aimed to fabricate, including their geometric design, piezoelectric film stacking, material selection, etc.

#### 2.3.1. Substrate Structural Design

**Figure 2:**
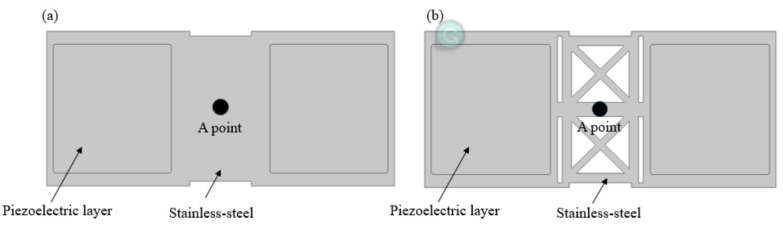
A comparison of the (**a**) full substrate and (**b**) etched substrate.

The vibration of a diaphragm creates a sound wave by changing pressure over time, which disturbs the air molecules near the diaphragm. As a result, the sound pressure level of a speaker is proportional to the amplitude of its diaphragm’s displacement. In this study, the substrate is actuated by a piezoelectric film. To maximize the deformation of the substrate, its structural design must be considered. Reducing the material at the center of the substrate to create an etched structure can improve its displacement amplitude. Figure 2 illustrates the two different substrate configurations considered for use in this study.

**Figure 3:**
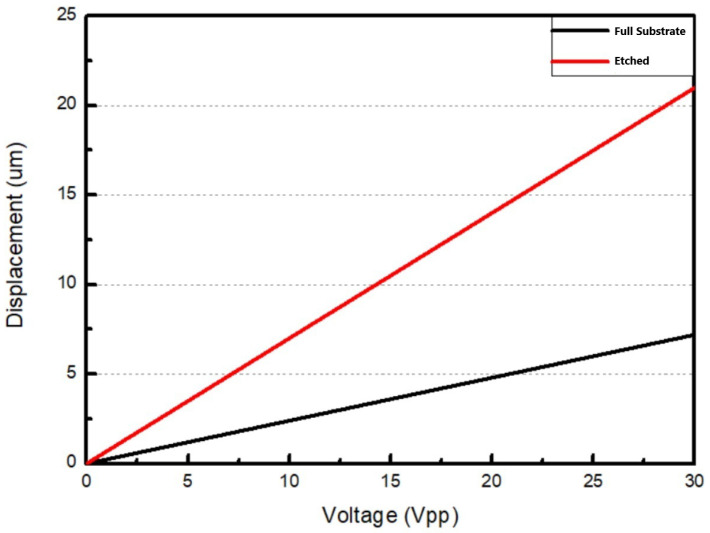
The difference in displacement at the center of the substrate (the A point in Figure 2).

From Figure 3, we can see that there is a fourfold difference in displacement amplitude between these substrates.

As a result, we use the etched design as the substrate candidate.

#### 2.3.2. Bimorph Single-Layer Configuration

**Figure 4:**
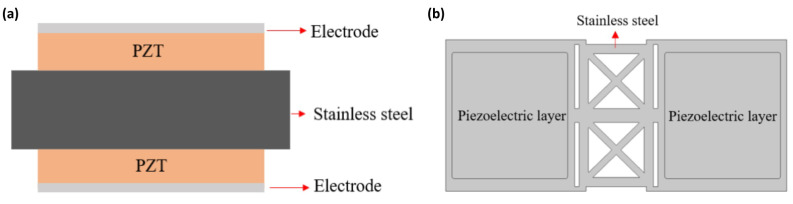
(**a**) The cross view and (**b**) the top view of the bimorph single-layer configuration speaker.

Having chosen an etched design for the substrate, we then chose a bimorph structure for our design, which is shown in Figure 4, in order to achieve a symmetric response to stress in the substrate and amplify the strain when a voltage is applied.

The substrate we used is a 30 µm thick stainless steel that is 12 mm in width and 6.8 mm in length. Two 10 µm thick PZT piezoelectric films were deposited using the aerosol deposition method we developed. Finally, this metal is used as an electrode and placed on top of the piezoelectric film.

**Figure 5:**
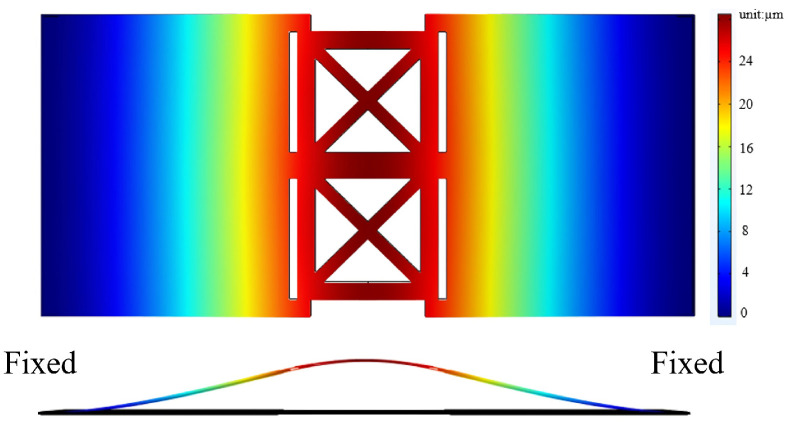
The displacement of the bimorph single-layer configuration at the substrate’s first resonant frequency.

In this study, we focus on the performance of microspeakers in their low-frequency range. That is, the modal analysis of their first resonance plays an important role in our analysis. Figure 5 illustrates the displacement distribution of the substrate at its first resonant frequency, with a fixed-fixed boundary condition set at the edge of the substrate. The first resonant frequency is located at 830.2 Hz, with a maximum displacement of around 28 µm seen at the center of the substrate.

#### 2.3.3. Bimorph Multi-Layer Configuration

**Figure 6:**
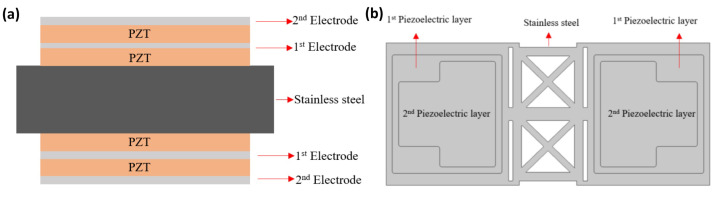
(**a**) The cross view and (**b**) the top view of the bimorph single-layer configuration speaker.

To achieve the same thickness in a piezoelectric film created with multi-layer stacking, we split the thickness of the piezoelectric film, changing it from a single 10 µm film into two 5 µm films to enable a multi-layer configuration. The design is shown in Figure 6.

**Figure 7:**
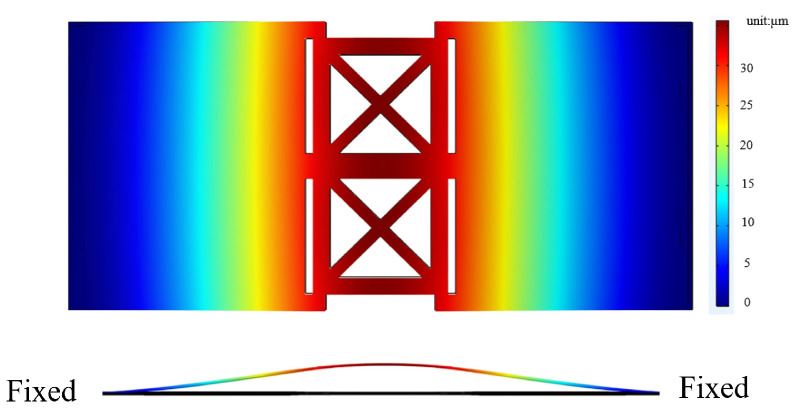
The displacement of the bimorph multi-layer configuration under the substrate’s first resonant frequency.

The results of our modal analysis are shown in Figure 7. For the multi-layer configuration, the first resonant frequency increases slightly to 895.4 Hz.

### 2.4. Acoustic Analysis of Piezoelectric Microspeaker

**Figure 8:**
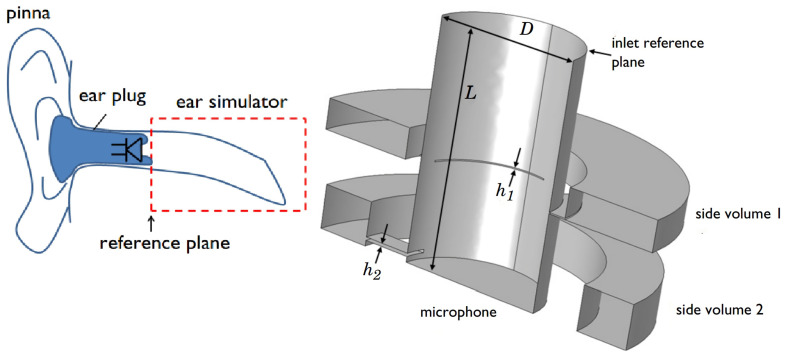
A schematic view of the human ear canal and a schematic view of the simulation we conducted [29].

To simulate the human ear canal, we used the standard (IEC 60318-4) [29], which details the simulation of enclosed-field acoustic conditions. Figure 8 presents a schematic view of the human ear canal and a schematic view of the simulation. The shape of the simulated ear canal comes from COMSOL software and follows the IEC 60318-4 standards, with acoustic properties that correspond to the Brüel & Kjær Ear Simulator Type 4157 Simulator (Brüel & Kjær, Nærum, Denmark). The values of D, L, $h_{1}$, and $h_{2}$ are 7.5 mm, 12.5 mm, 69 µm, and 170 µm, respectively.

The ear canal simulation includes one end that works as an inlet reference plane and several side volumes that comply with the standards we followed. A microphone is placed at the other end of the canal to simulate the human eardrum, which receives pressure differences.

**Figure 9:**
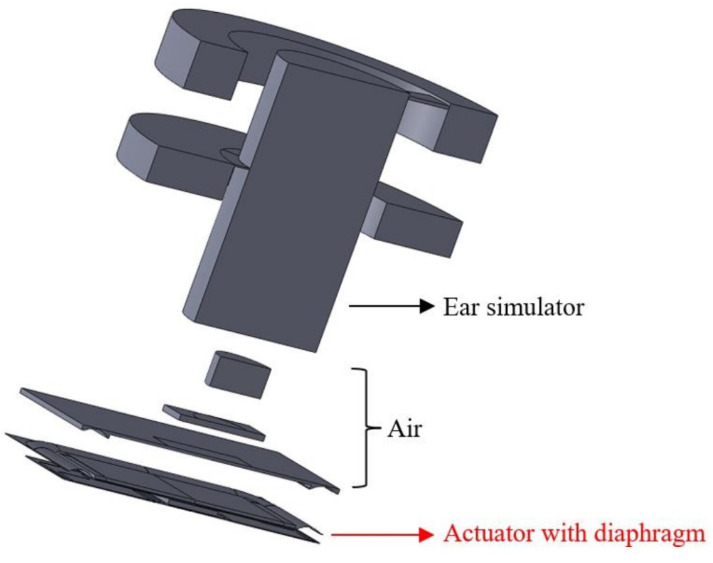
Exploded view of the simulated ear canal and designed speaker.

**Figure 10:**
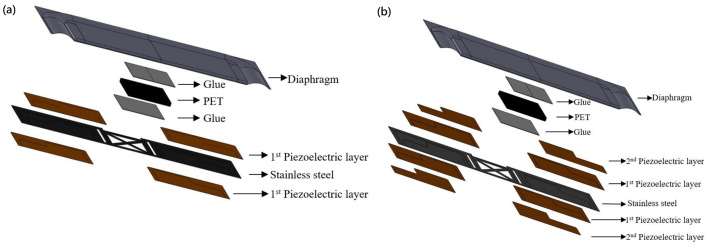
Exploded view of the speakers: (**a**) bimorph single-layer configuration and (**b**) bimorph multi-layer configuration.

Combining this with the design mentioned in the previous section, Figure 9 and Figure 10 present exploded views of the simulated ear canal and designed speaker to fully elucidate the simulation conditions we used.

The diaphragm and coupling proof mass are bonded on top of the electrode so that they can be driven by the inverse piezoelectric effect. The driving voltages are set at 15 Vpp and 30 Vpp, with a frequency that ranges from 0 Hz to 1 kHz. Table 1 lists the geometric dimensions of the bimorph single-layer and the multi-layer microspeakers.

## 3. Microspeaker Fabrication Process

### 3.1. Aerosol Deposition Method

The fabrication process we used is a combination of the aerosol deposition method and the traditional metal MEMS fabrication process. In this study, the most critical part of the device is the high-quality piezoelectric film that is deposited on the substrate and in stacked layers, if needed [30,31,32,33].

**Figure 11:**
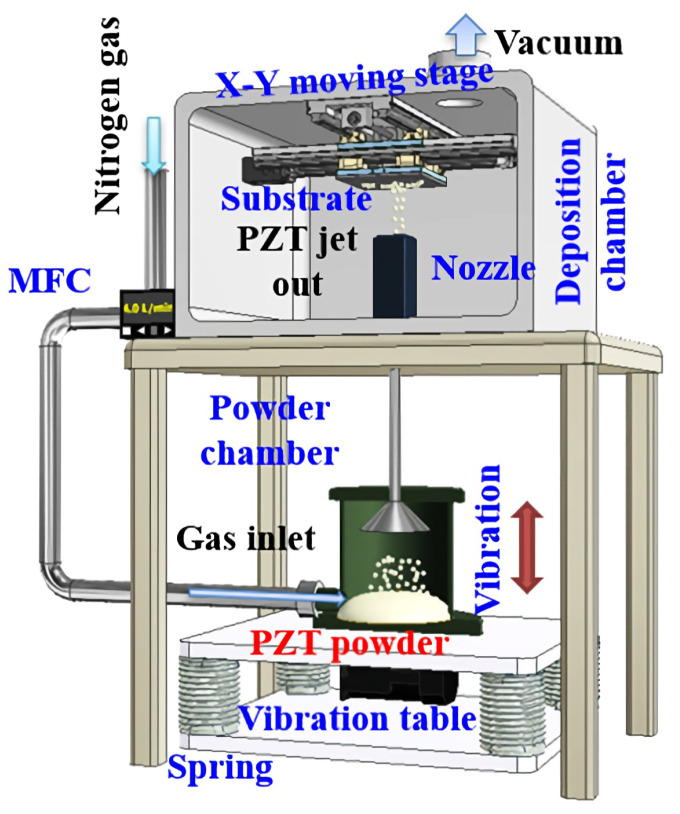
A schematic view of the aerosol deposition machine [30].

The aerosol deposition machine we developed contains two chambers, as shown in the schematic in Figure 11.

The upper chamber is under vacuum, with an X-Y moving stage installed inside the chamber to deposit the material more evenly. The lower chamber is filled with a piezoelectric material powder and high-pressure nitrogen gas, which are mixed using the vibration table. The pressure difference creates a high-speed air stream that carries the piezoelectric material through the nozzle to deposit it on the substrate. By tuning the mass flow controller, the moving stage, and the duration of the deposition, the thickness of the piezoelectric film can be carefully controlled.

### 3.2. Metal Micro-Electrical–Mechanical System (MEMS) Process

**Figure 12:**
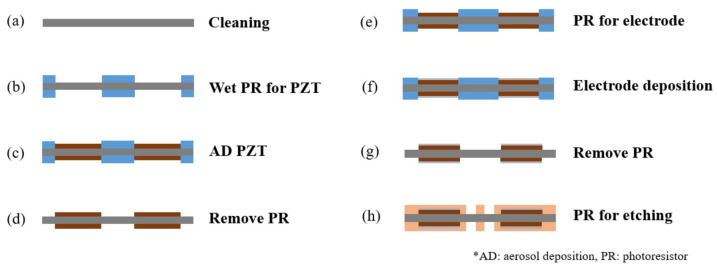
The process of creating the bimorph single-layer configuration.

**Figure 13:**
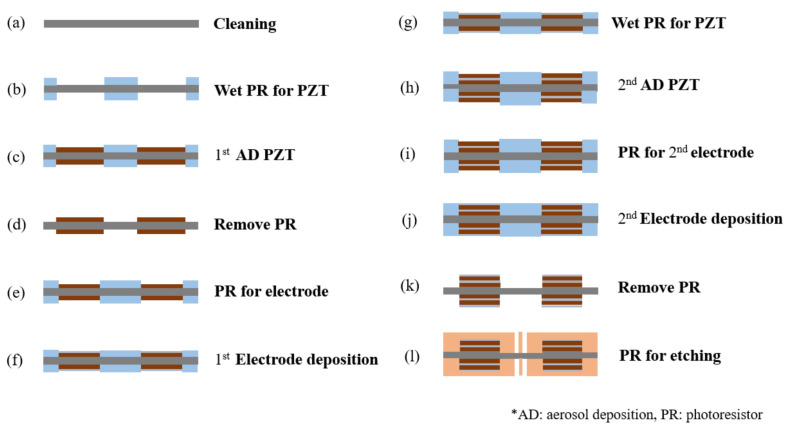
The process of creating the bimorph multi-layer configuration.

After verifying the design of the microspeaker using the COMSOL simulation software, the metal MEMS process was used to fabricate the device. Figure 12 and Figure 13 show the fabrication process of two the microspeaker configurations mentioned in the previous section. First, 30 µm thick stainless steel was cleaned with piranha solution. Using the designed mask and negative photoresist, the PZT deposition area was defined on the substrate. The PZT film was deposited through the aerosol deposition method and the films were removed from the photoresist through a lift-off process. For the bimorph single-layer configuration, the PZT film was deposited with a thickness of 10 µm on each side. For the bimorph multi-layer configuration, the PZT film was deposited with a thickness of 5 µm on each side. The electrode pad was deposited through an e-beam evaporator and was made up of 200 nm of platinum on top of 20 nm of titanium. To create the bimorph multi-layer configuration, the process was run again to deposit the second layer of the PZT film and the electrode. As the last step, the devices were released from the substrate by wet etching them with aqua regia.

### 3.3. Annealing and Poling

**Figure 14:**
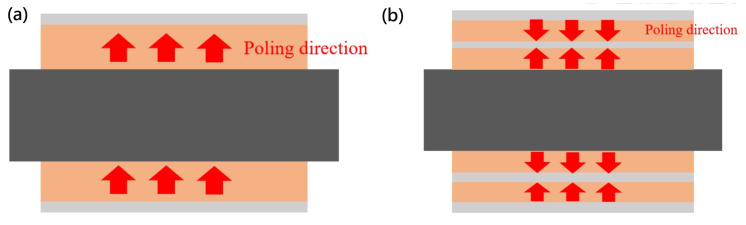
A schematic view the poling connection used in (**a**) bimorh single-layer and (**b**) bimorph multi-layer configurations.

**Figure 15:**
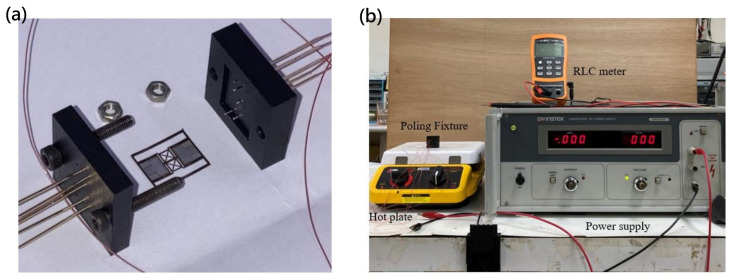
(**a**) The fixture clamp used for poling (with the actuator in between) and (**b**) the setup for poling process.

After the release process, the devices were annealed at below 650 °C for 24 h in a furnace and then cooled to room temperature. This poling process is necessary for enhancing the piezoelectric characteristics of a device and reducing residual stress from the metal MEMS process. During the annealing process, the electric dipole moment may be lost and randomized. To re-align the electric dipole moment, the devices were put on a hot plate which was connected to a power supply with a sufficient voltage difference. The PZT layers were poled at 150 °C and a DC voltage of 120 V. Figure 14 illustrates the poling setup for the bimorph single-layer and multi-layer configuration, respectively, while Figure 15 illustrates the poling clamp and the poling setup.

### 3.4. Packaging

**Figure 16:**
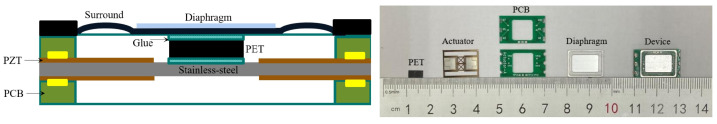
The cross-view of the package and the dimensions of each of its parts.

Finally, the devices were created by packaging the actuators with the diaphragm; PET, which acts as the coupling mass; and the printed circuit board (PCB). The adhesion process was carried out using commercial adhesive epoxy. Figure 16 illustrates the cross-view of the package and the dimensions of each part of the package. The dimensions of the speakers, when combined with the printed circuit board, are 20.0 × 12.0 × 3.0 mm^3^.

## 4. Results and Discussion

**Figure 17:**
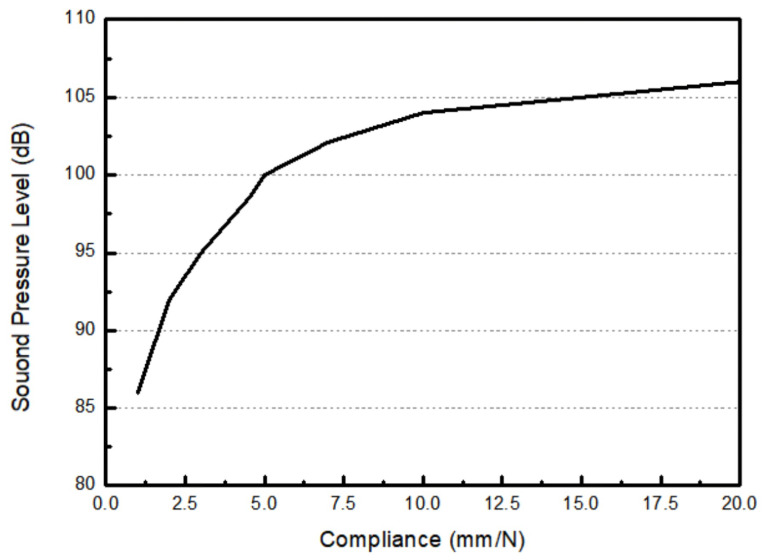
The simulated results of the relationship between compliance and sound pressure level in a bimorph multi-layer configuration speaker.

The diaphragm is made up of three parts—the frame, cap, and edge—and its purpose is to disturb the air to generate a change in pressure. According to the study in [34], the compliance value of the edge of the diaphragm plays an important role in maintaining the rebound force to within reasonable limits during driving behavior. Ideally, a diaphragm with a higher edge compliance value performs better. To address the sound pressure level performance of diaphragm under different compliance values, we used our ear canal simulation to evaluated our bimorph multi-layer configuration speaker. The driving frequency was set at 1 kHz and the driving voltage was set at 30 Vpp. The simulation results in Figure 17 illustrate that the sound pressure level is insufficient when the compliance value is below 5 mm·N^−1^ due to the heavy load on the actuator. On the other hand, the sound pressure level can reach more than 100 dB when the compliance of the diaphragm is greater than at least 7.5 mm·N^−1^. Therefore, we set the compliance value threshold to 10 mm·N^−1^ for the outsourced diaphragm.

To examine whether the outsourced diaphragm matched the threshold we set, we measured the difference in displacement seen when different weights were connected to the diaphragm using a laser displacement meter. The compliance constant is the reciprocal value of the spring constant, which can be derived from Hooke’s law (Equation (5)):

(5) $$k=\frac{F}{x}=\frac{mg}{x}$$

**Figure 18:**
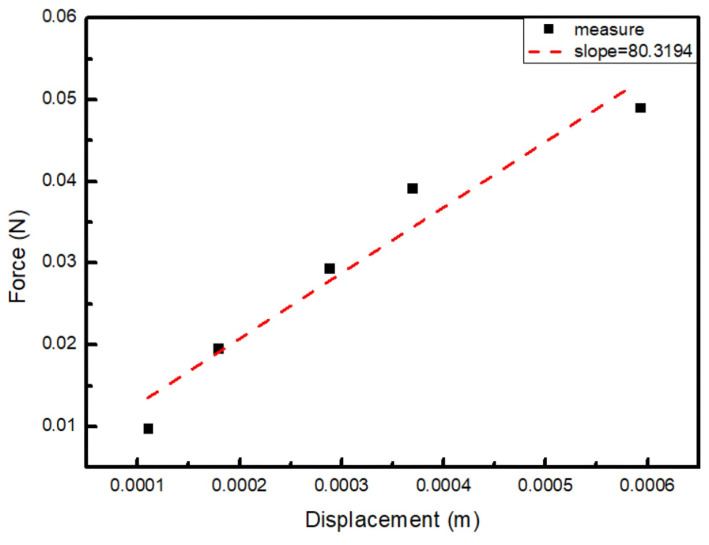
The relationship between the force applied and the displacement of the diaphragm.

Table 2 illustrates the measured compliance values of the sample diaphragm, which were greater than 10 mm·N^−1^ and quite closely matched the values that we expected under different loads. From Figure 18, using linear regression, we can see that the reciprocal value of the slope is 12.45 mm·N^−1^, which is the compliance value of the diaphragm, meaning it is suitable for use in the final packaging that will undergo acoustic measurements.

**Figure 19:**
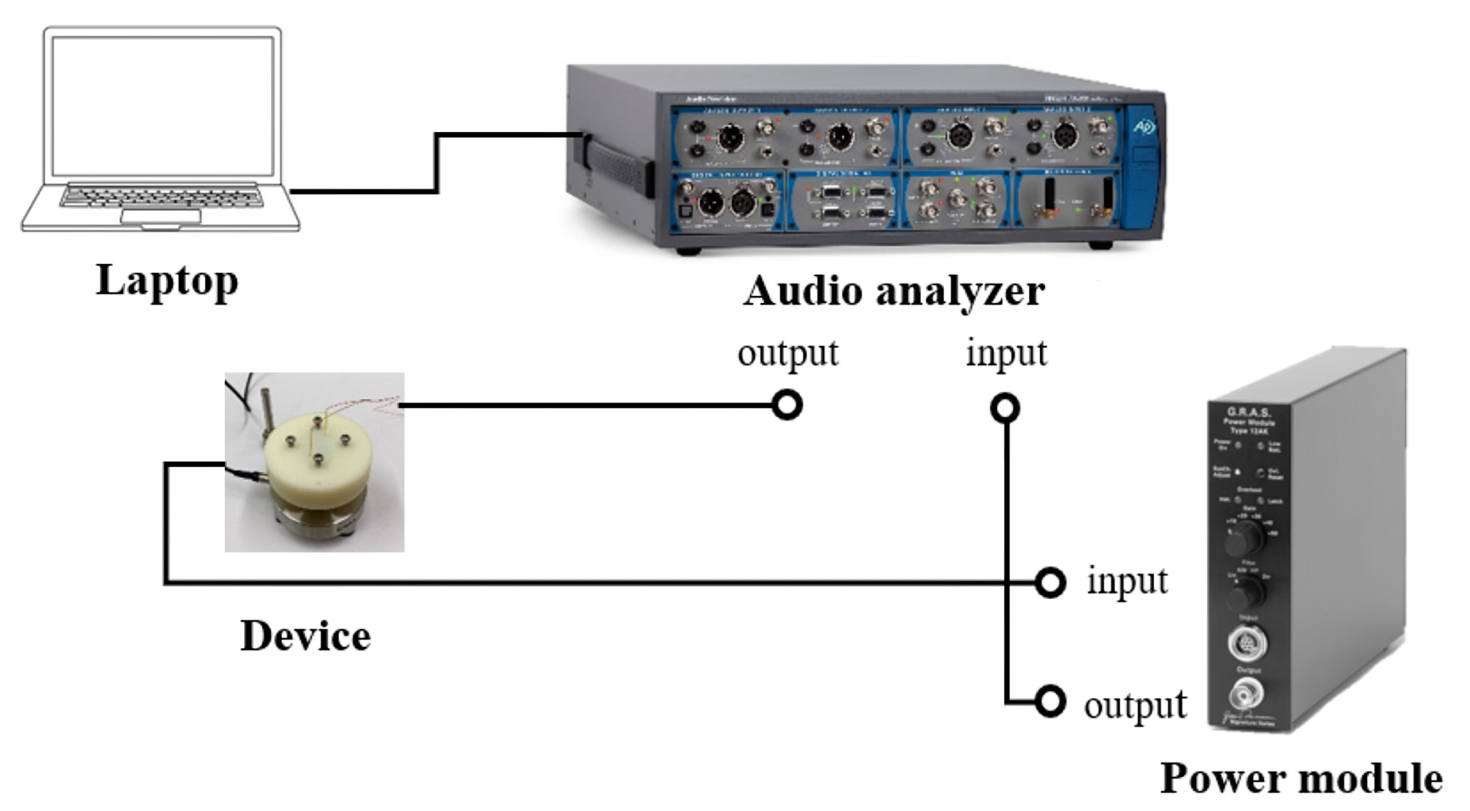
A schematic view of the enclosed-field acoustic measurement.

**Figure 20:**
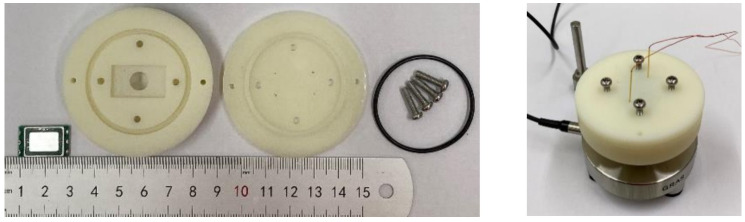
The clamp for the speaker alone and combined with the ear canal simulator.

The setup used for the acoustic analysis is shown in Figure 19. A laptop is connected to an audio analyzer (APx525 Audio Analyzer, Audio Precision, Beaverton, OR 97008, USA) to control the measurement sequences. The audio analyzer sends the signal to a power module to amplify the driving voltage to activate our device. Finally, the results are fed back to the audio analyzer for further analysis. Figure 20 illustrates the ear canal simulator (RA0045, GRAS Sound & Vibration, Holte, Denmark), which is combined with a clamp for enclosed acoustic analysis.

**Figure 21:**
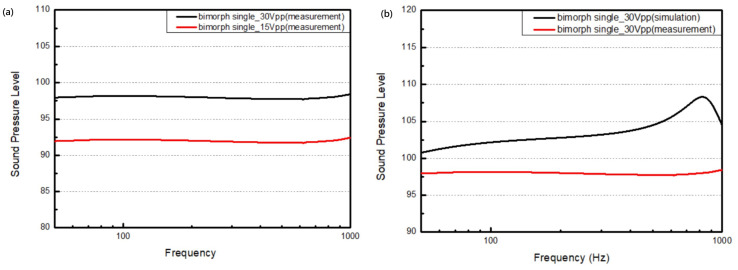
The output performance of the microspeaker with a bimorph single-layer configuration (**a**) under different driving voltages and (**b**) in comparison to its output as simulated under 30 Vpp.

This study focuses mainly on the results of the speakers in the low-frequency range. As a result, we set the frequency range to sweep from 0 Hz to 1 kHz. The driving voltages were set to 30 Vpp and 15 Vpp. Figure 21a illustrates the sound pressure level and the frequency change for the bimorph single-layer configuration. It can be seen that the sound pressure level is proportional to the driving voltage. Under the 15 Vpp condition, the sound pressure level stays above 91.4 dB, with a maximum value of 92.4 dB. There is about a 6 dB difference under the 30 Vpp condition, where the sound pressure level remains above 97.4 dB, with a maximum value of 98.4 dB.

Figure 21b illustrates the difference between the simulation and measurements from the real device with a bimorph single-layer configuration. Compared to the simulation, there is no obvious peak in the sound pressure level at the first resonant frequency, i.e., 830.2 Hz. There are differences of approximately 5.5 dB in the sound pressure level and in the measured frequency range, with a maximum value of 10.2 dB the resonant frequency.

**Figure 22:**
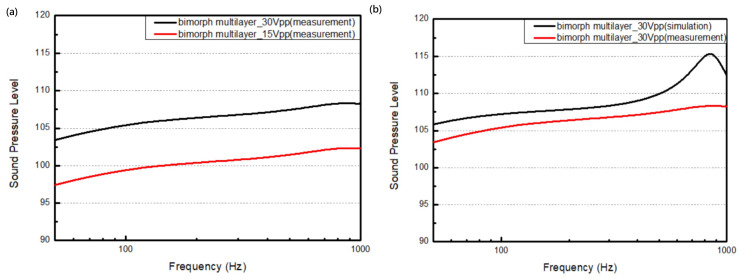
The output performance of the microspeaker with a bimorph multi-layer configuration (**a**) under different driving voltages and (**b**) in comparison to its output as simulated under 30 Vpp.

For the bimorph multi-layer configuration, we obtained the same performance trends as those seen with the bimorph single-layer configuration. The higher the driving voltage we apply, the higher the sound pressure level we obtain from the device. Figure 22a illustrates the differences in sound pressure level of bimorph multi-layer configuration under different driving voltages. Under the 15 Vpp condition, the sound pressure level remains above 97.4 dB, with a maximum value of 102.2 dB. There is about a 5 dB difference under the 30 Vpp condition, for which the sound pressure level remains above 103.4 dB, with a maximum value of 108.2 dB.

Figure 22b illustrates the difference between the simulation and measurements from the real device with the bimorph multi-layer configuration. When compared to the simulation, there is still no obvious peak in the sound pressure level at the first resonant frequency, i.e., 895.4 Hz. There are differences of approximately 2 dB in the sound pressure level and the measurement frequency range, with a maximum value of 7.1 dB at the resonant frequency.

**Figure 23:**
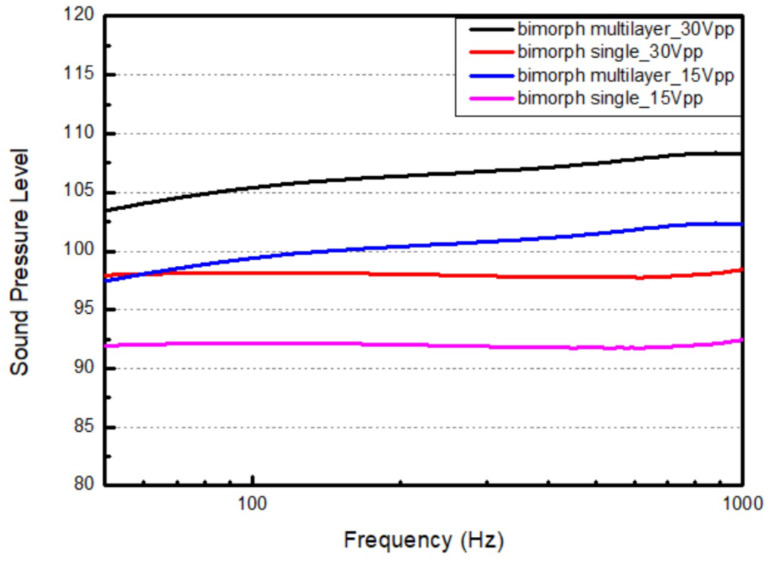
The comparison of two bimorph configurations under different driving voltages.

From the results shown above, we can first improve the displacement of the substrate by reducing its mass. Second, the sound pressure level follows the same trend but at different values when the same speaker configuration is subjected to different driving voltages. Third, we discovered from the measurement results that both the bimorph single-layer (BSL) configuration and the bimorph multi-layer (MBL) configuration have a flat response rather than a peak response. We consider this to be caused by the higher damping effect of the glue bonding in real life than in the device we simulated, where we used the default material parameters from simulation tool library for the diaphragm. In the FEM modeling, we shaped the design of the diaphragm with the default material and mechanical parameters from the simulation tool we used. Also, the real sample diaphragm has a microstructure around its edge that we were hardly able to take into account due to the complex design created by the company, as it may have different properties compared to those in the default material library. Lastly, the bimorph multi-layer (MBL) configuration speaker obtains a better sound pressure level due to the higher electric field intensity across its piezoelectric film, which generates larger deformation and enables a larger displacement of the diaphragm. In other words, as seen in Figure 23, we can apply a lower driving voltage to the bimorph multi-layer configuration to obtain almost the same performance as that of the bimorph single-layer configuration, which has the same overall thickness to its piezoelectric film. In this study, we may take advantage of the diaphragm area to create a sound pressure level that is higher than that in the works of others. However, the more bulky the speakers, the more force must be created to push the structures to meet the requirement. We strike the balance in device design by keeping the speakers in reasonable size with thicker piezo-layers, but still obtain a good performance result.

Table 3 lists two of the existing types of MEMS microspeakers: whole MEMS and piezo-actuated microspeakers. The main difference between the two is the frequency of their maximum SPL. From the table, it can be seen that their frequency points are higher than 1 kHz, which means they can be used as tweeter units. In this study, the woofer unit of a piezo-actuated MEMS speaker is realized.

## 5. Conclusions

In this study, we developed two configurations for piezoelectric microspeakers that allow for the creation of a woofer-type unit. This is in contrast to other research, which has focused mainly on tweeter-type units.

To amplify the displacement of the diaphragm under the substrate’s first resonance frequency, the substrate was etched with a specific pattern to reduce its mass and rigidity for better flexibility. This resulted in a 4 × larger displacement.

To create the substrate, we deposited a high-quality piezoelectric film on structurally designed stainless steel using the aerosol deposition method we developed in previous work. Compared to the bimorph single-layer configuration, which features a thick layer of PZT film on both sides, the bimorph multi-layer configuration reduces the thickness of the PZT film by half and then stacks another half-thickness PZT film on top to create the same total thickness. We believe that the thinner PZT film receives the electric field more evenly and is less rigid, enabling it to deformation further under driving compared to a single thick layer. The devices’ fabrication was completed using the standard metal MEMS fabrication process to maintain cost effectiveness and enable mass production.

After finishing the actuator, the module was fully packaged with PET, which was used as the coupling mass; a diaphragm; and a printed circuit board to form a full microspeaker. From the results of simulations based on our actuator design, the compliance value of the edge of the diaphragm needs to be greater than a specific value to enable the best sound pressure level performance. In our design, the edge’s compliance value should be greater than 10 mm·N^−1^.

An enclosed-field acoustic analysis was performed with an IEC-60318-4 standard ear canal simulator. The sound pressure level of the bimorph single-layer configuration shows a flat response below 1 kHz. Additionally, the maximum values of the sound pressure level are 92.4 dB and 98.4 dB under applied voltages of 15 Vpp and 30 Vpp, respectively. On the other hand, for the part with a bimorph multi-layer configuration, the film thickness can be controlled to within 8 % when depositing four 5 µm piezoelectric film layers while maintaining structural symmetry. Its frequency response shows the same trend with the two applied voltages. The maximum values of the sound pressure level are 102.2 dB and 108.2 dB under applied voltages of 15 Vpp and 30 Vpp, respectively.

Finally, we have shown that the bimorph multi-layer configuration can reach a similar sound pressure level performance to that of the bimorph single-layer configuration but with a lower driving voltage. These results demonstrate that this work has improved the sound pressure level performance of microspeakers in their low-frequency range.

## 6. Patents

This work has a patent registered at the Intellectual Property Office, Ministry of Economic Affairs, Republic of China (Taiwan), with the number TW202402068A. 

## Figures and Tables

**Table 1:** Geometric dimensions of the bimorph single-layer and multi-layer microspeakers.

Parameter	Single Bimorph Layer	Multiple Bimorph Layers
Stainless Steel Thickness	30 µm	30 µm
Stainless Steel Dimension	12 × 6.8 mm^2^	12 × 6.8 mm^2^
No. of Piezoelectric Layer	2	4
Piezoelectric Film Thickness per Layer	10 µm	5 µm
Total Piezoelectric Film Thickness	20 µm	20 µm

**Table 2:** The compliance value of the diaphragm, measured by a laser displacement meter.

Weight (g)	Force (N)	Displacement (m)	k (N/m)	Compliance (mm/N)
1	0.00981	0.000110	89.29	11.2
2	0.01962	0.000179	109.89	9.1
3	0.02943	0.000288	102.05	9.8
4	0.03924	0.000369	106.38	9.4
5	0.04905	0.000593	82.67	12.1

**Table 3:** Comparison of existing piezoelectric microspeakers.

Reference	Material	Piezo-Layer Thickness	Diaphragm Size	Maximum SPL	Distance	Driving Voltage
Fully MEMS (without diaphragm)						
[20]	Sputter PZT	1 µm	4×4 mm^2^	106.8 dB @ 1.85 kHz	^*a*^	15 Vpp
[23]	ZnO	0.5 µm	3×3 mm^2^	83.1 dB @ 13.3 kHz	10 mm	30 Vpp
[24]	AlN	0.5 µm	4×4 mm^2^	100 dB @ 10.0 kHz	3 mm	20 Vpp
[25]	Sol–gel PZT	2 µm	1×1 mm^2^	110 dB	^*a*^	20 Vpp
[35]	Ceramic PZT	5 µm	ϕ 6 mm	119 dB @ 9 kHz	10 mm	10 Vpp
Piezoelectric Actuated (with diaphragm)						
[27]	Unknown	Unknown	6.7×4.7 mm^2^	52 dB @ 1 kHz	^*b*^	15 VDC +15 Vpp
This work (BSL) ^*c*^	Aerosol PZT	20 µm	15×12 mm^2^	98.4 dB @ 830.2 Hz	^*a*^	30 Vpp
This work (BML) ^*d*^	Aerosol PZT	20 µm	15×12 mm^2^	108.1 dB @ 895.4 Hz	^*a*^	30 Vpp

^*a*^: tested under the IEC 60318-4 standard; ^*b*^: tested under the IEC 60268-5 standard; BSL ^*c*^: bimorph single-layer; BML ^*d*^: bimorph multi-layer.

## Data Availability

The data presented in this study are available on request from the corresponding author.

## References

[1] Status of the MEMS Industry 2021. https://medias.yolegroup.com/uploads/2021/07/YINTR21180-Status-of-the-MEMS-Industry-2021_Sample.pdf.

[2] Microphones, Microspeakers, and Audio Processing 2021 Report. https://www.yolegroup.com/product/report/microphones-microspeakers-and-audio-processing-2021/.

[3] Cheng M.-C., Huang W.-S., Huang S.R.-S. (2004). A silicon microspeaker for hearing instruments. J. Micromech. Microeng..

[4] Harradine M., Birch T., Stevens J., Shearwood C. A micro-machined loudspeaker for the hearing impaired. Proceedings of the International Solid State Sensors and Actuators Conference (Transducers’ 97).

[5] Je S.-S., Wang N., Brown H.C., Arnold D.P., Chae J. An electromagnetically actuated microspeaker with fully-integrated wax-bonded Nd-Fe-B micromagnets for hearing aid applications. Proceedings of the TRANSDUCERS 2009-2009 International Solid-State Sensors, Actuators and Microsystems Conference.

[6] Lemarqu G., Ravaud R., Shahosseini I., Lemarqu V., Moulin J., Lefeuvre E. (2012). MEMS electrodynamic loudspeakers for mobile phones. Appl. Acoust..

[7] Rashedin R., Meydan T., Borza F. (2006). Electromagnetic micro-actuator array for loudspeaker application. Sens. Actuators A Phys..

[8] Setiarini A., Sugandi G., Wijayanto Y.N., Wiranto G., Manurung R.V., Hermida I.D.P. A novel structure of electromagnetic mems speaker for hearing aid application. Proceedings of the 2018 International Conference on Radar, Antenna, Microwave, Electronics, and Telecommunications (ICRAMET).

[9] Chiang H.-Y., Huang Y.-H. (2015). Vibration and sound radiation of an electrostatic speaker based on circular diaphragm. J. Acoust. Soc. Am..

[10] Huang Y.-H., Chiang H.-Y. (2016). Vibrational mode and sound radiation of electrostatic speakers using circular and annular diaphragms. J. Sound Vib..

[11] Kaiser B., Langa S., Ehrig L., Stolz M., Schenk H., Conrad H., Schenk H., Schimmanz K., Schuffenhauer D. (2019). Concept and proof for an all-silicon MEMS micro speaker utilizing air chambers. Microsyst. Nanoeng..

[12] Roberts R.C., Du J., Ong A.O., Li D., Zorman C.A., Tien N.C. (2007). Electrostatically driven touch-mode poly-SiC microspeaker. Sensors.

[13] Tumpold D., Stark M., Euler-Rolle N., Kaltenbacher M., Jakubek S. (2015). Linearizing an electrostatically driven MEMS speaker by applying pre-distortion. Sens. Actuators A Phys..

[14] Zhou Q., Zettl A. (2013). Electrostatic graphene loudspeaker. Appl. Phys. Lett..

[15] Bobinger M., La Torraca P., Mock J., Becherer M., Cattani L., Angeli D., Larcher L., Lugli P. (2018). Solution-processing of copper nanowires for transparent heaters and thermo-acoustic loudspeakers. IEEE Trans. Nanotechnol..

[16] Fei W., Zhou J., Guo W. (2015). Low-voltage Driven Graphene Foam Thermoacoustic Speaker. Small.

[17] Liu Y., Tong L., Lai S.K. (2018). Thermo-acoustics generated by periodically heated thin line array. J. Sound Vib..

[18] Wang D., He X., Zhao J., Jin L., Ji X. (2020). Research on the electrical-thermal-acoustic conversion behavior of thermoacoustic speakers based on multilayer graphene film. IEEE Sens. J..

[19] Casset F., Dejaeger R., Laroche B., Desloges B., Leclere Q., Morisson R., Bohard Y., Goglio J.P., Escato J., Fanget S. (2015). A 256 MEMS membrane digital loudspeaker array based on PZT actuators. Procedia Eng..

[20] Cheng H.-H., Huang Z.-R., Wu M., Fang W. Low frequency sound pressure level improvement of piezoelectric MEMS microspeaker using novel spiral spring with dual electrode. Proceedings of the 20th International Conference on Solid-State Sensors, Actuators and Microsystems & Eurosensors XXXIII (TRANSDUCERS & EUROSENSORS XXXIII).

[21] Cho I.-J., Jang S., Nam H.-J. (2009). A piezoelectrically actuated mems speaker with polyimide membrane and thin film pb (zr, ti) o3 (pzt) actuator. Integr. Ferroelectr..

[22] Kim H., Yang W. (2015). The effects of electrodes patterned onto the piezoelectric thin film on frequency response characteristics of PMN-PT MEMS acoustic actuators. J. Electroceram..

[23] Ko S.C., Kim Y.C., Lee S.S., Choi S.H., Kim S.R. (2003). Micromachined piezoelectric membrane acoustic device. Sens. Actuators A Phys..

[24] Seo K., Park J., Kim H., Kim D., Ur S., Yi S. (2007). Micromachined piezoelectric microspeakers fabricated with high quality AlN thin film. Integr. Ferroelectr..

[25] Stoppel F., Eisermann C., Gu-Stoppel S., Kaden D., Giese T., Wagner B. Novel membrane-less two-way MEMS loudspeaker based on piezoelectric dual-concentric actuators. Proceedings of the 19th International Conference on Solid-State Sensors, Actuators and Microsystems (TRANSDUCERS).

[26] Yi S., Ur S.C., Kim E.S. Performance of packaged piezoelectric microspeakers depending on the material properties. Proceedings of the IEEE 22nd International Conference on Micro Electro Mechanical Systems.

[27] ADAP UT-P2017 Specification. https://www.usound.com/wp-content/uploads/2019/05/2019-05_Adap_UT-P-2017_Datasheet.pdf.

[28] (2010). Electroacoustics—Simulators of Human Head and Ear, Edition 1.0.

[29] Wille M., Rasmussen P. IEC 60318-4 ear simulator for low noise measurements & anthropometric rubber pinna. Proceedings of the Audio Engineering Society Conference: 2016 AES International Conference on Headphone Technology.

[30] Lin S.C., Wu W.J. (2013). Fabrication of PZT MEMS energy harvester based on silicon and stainless-steel substrates utilizing an aerosol deposition method. J. Micromech. Microeng..

[31] Kuo C.L., Lin S.C., Wu W.J. (2016). Fabrication and performance evaluation of a metal-based bimorph piezoelectric MEMS generator for vibration energy harvesting. Smart Mater. Struct..

[32] Kuo Y.C., Chien J.T., Shih W.T., Chen C.T., Lin S.C., Wu W.J. The fatigue behavior study of micro piezoelectric energy harvester under different working temperature. Proceedings of the SPIE 10967, Active and Passive Smart Structures and Integrated Systems XIII.

[33] Gong X., Kuo Y.C., Zhou G., Wu W.J., Liao W.H. (2023). An aerosol deposition based MEMS piezoelectric accelerometer for low noise measurement. Microsyst. Nanoeng..

[34] Kim W., Jang G.-W., Kim Y.Y. (2009). Microspeaker diaphragm optimization for widening the operating frequency band and increasing sound pressure level. IEEE Trans. Magn..

[35] Wang H., Chen Z., Xie H. (2020). A high-SPL piezoelectric MEMS loud speaker based on thin ceramic PZT. Sens. Actuators A Phys..

